# Evaluating the link between hearing loss and Alzheimer’s disease neuropathology: A systematic review and meta‐analysis

**DOI:** 10.1002/alz.092211

**Published:** 2025-01-03

**Authors:** Avinash Chandra, Benjamin Aaron Levett, Sheena Waters, Chris JD Hardy, Jason D Warren, Charles R Marshall

**Affiliations:** ^1^ Queen Mary University of London, London United Kingdom; ^2^ Dementia Research Centre, UCL Queen Square Institute of Neurology, University College London, London, London United Kingdom; ^3^ Dementia Research Centre, Department of Neurodegenerative Disease, UCL Queen Square Institute of Neurology, University College London, London United Kingdom; ^4^ Dementia Research Centre, UCL Queen Square Institute of Neurology, University College London, London United Kingdom

## Abstract

**Background:**

Various explanations have been proposed for how hearing impairment might be associated with increased risk of dementia. Several theories have proposed direct links with Alzheimer’s disease (AD) neuropathology, either due to shared aetiology (i.e. AD proteinopathies cause impaired hearing), or due to an interaction (i.e. functional brain changes due to impaired hearing drive pathogenic protein accumulation). We performed a systematic literature review and meta‐analysis to evaluate evidence associating AD neuropathology (amyloid‐β and tau) with hearing loss.

**Method:**

Searches were conducted using electronic biomedical databases for articles published in the past 20 years. Studies evaluating hearing loss as an exposure and AD neuropathology using molecular biomarkers or histopathology as an outcome were included. Initial search identified 7,313 studies, which were screened, independently reviewed, and assessed for quality before being synthesised and meta‐analysed.

**Results:**

Studies were highly heterogeneous, with mixed and inconsistent results. There was overall no strong evidence linking hearing loss with amyloid‐ β pathology, with some studies reporting weak effect sizes and others null results. Associations between hearing and tau pathology were more robust. Associations between hearing and neuropathology were stronger when hearing impairment was measured using more “central” tests of hearing than standard “peripheral” measures such as pure tone audiometry, suggesting that changes in the auditory brain may be a consequence of AD neuropathology.

**Conclusion:**

There is no strong evidence to suggest that changes in peripheral hearing are associated with AD neuropathology. Tests of central auditory function may signal AD neuropathological changes in auditory cortical areas. These results suggest the need for caution in developing hearing‐targeted interventions, such as hearing aid use, for prevention of neuropathological change due to Alzheimer’s disease.

